# Unsupervised Neural Network Quantifies the Cost of Visual Information Processing

**DOI:** 10.1371/journal.pone.0132218

**Published:** 2015-07-22

**Authors:** Levente L. Orbán, Sylvain Chartier

**Affiliations:** School of Psychology, University of Ottawa, Ottawa, Ont., Canada; University of Arizona, UNITED STATES

## Abstract

Untrained, “flower-naïve” bumblebees display behavioural preferences when presented with visual properties such as colour, symmetry, spatial frequency and others. Two unsupervised neural networks were implemented to understand the extent to which these models capture elements of bumblebees’ unlearned visual preferences towards flower-like visual properties. The computational models, which are variants of Independent Component Analysis and Feature-Extracting Bidirectional Associative Memory, use images of test-patterns that are identical to ones used in behavioural studies. Each model works by decomposing images of floral patterns into meaningful underlying factors. We reconstruct the original floral image using the components and compare the quality of the reconstructed image to the original image. Independent Component Analysis matches behavioural results substantially better across several visual properties. These results are interpreted to support a hypothesis that the temporal and energetic costs of information processing by pollinators served as a selective pressure on floral displays: flowers adapted to pollinators’ cognitive constraints.

## Introduction

Cognitive systems were shaped by selective pressures to minimize costs related to temporal delay and energetic requirements of information processing [1, 2]. These broad pressures have shifted organisms to use as little and as relevant information as possible to make fast and optimal choices in an uncertain environment [3–6]. However, even strong selective pressures cannot push organisms to function beyond the boundaries of the physical laws that govern information processing, or their performance envelopes [7]. The purpose of the models here is to quantify the computational cost of several visual properties, and to translate these findings into empirically testable hypotheses with respect to bumblebee floral choice behaviour. In essence, we are seeking the performance envelope of visual information processing.

The results of several neural network models suggest that symmetry preference is a by-product of the brain’s learning dynamics, which are shaped by pressures to minimize information processing [8, 9]. Symmetry has the property that a large proportion of information (ie, approximately half in the case of bilateral symmetry) can be discarded without any information loss. Flowers may have been pressured by pollinators to produce visual displays that made it easier for pollinators to process them.

One model suggests that animals learn to prefer symmetry as a result of generalizing knowledge of objects to their various forms (ie, translations, rotations, transformations of a known object) [10]. A feed-forward network using a genetic algorithm illustrated this idea by training the network on artificial birds. The artificial birds consisted of a 6 × 6 pixel images that resembled birds with short or long tail lengths. Results showed that the network activated with longer tailed and longer winged patterns, but not tail-less or random patterns. Further mutation of the network produced a progressively increasing preference for exaggerated features. A property of these preferred exaggerated features is that they were bilaterally symmetric. Symmetry may be viewed as a form of exaggeration of non-symmetric variants.

Preference for symmetry also emerged in two identical feed-forward neural networks in which both models were trained to recognize bilaterally symmetric bird-tails of varying levels of fluctuating asymmetry [9]: one network exposed to a perfectly bilaterally symmetric pattern, whereas the other network exposed to imperfect variations. After training, the two trained networks did not differ in their preferences for bilaterally symmetric patterns. Therefore, the network that was not exposed to symmetric patterns generalized to symmetric patterns during training on different forms of imperfect variations.

While both of these neural networks imply that symmetry preference emerges as a by-product of visual processing, the feedforward genetic network indicates that symmetry preference should be an unlearned preference, and the second feedforward network implies that symmetry preference is a result of functional experience with imperfectly symmetrical floral patterns. The empirical literature on symmetry preferences has been also mixed: some studies showed symmetry preference as an unlearned capacity, but others showed symmetry as a learned ability. Our goal is to implement models that (1) test the symmetry preference as an unlearned capacity hypothesis, and (2) produce concrete, empirically verifiable predictions. The results of the models will be compared with experimental results of behavioural studies published elsewhere.

### Bumblebees as a Model of Information Processing

Bumblebees are an ideal model to study symmetry preferences, and information processing in general, due to their simpler but very capable brains [11]. Many behavioural studies have focussed on studying the kinds of visual characteristics of a flower that enable bumblebees and honeybees to identify them as a potential food source. Preferences for symmetry, along with properties such as shape, colour and background-foreground characteristics have been studied extensively. Studies typically deconstruct flowers into their constituents and choice behaviour is recorded at these flower-like visual properties by pitting two or more artificial flower patterns against one another [12].

Unlearned preferences for visual properties have been studied using flower naïve bumblebees, meaning that they have not received any rewards in a testing environment. Unlearned and unrewarded preferences have been observed for colours [13], shapes [14], symmetry [15], foliage background complexity [16], and pattern positioning [17]. For example, yellow and blue colours, nectar guides (i.e. radial, sunburst pattern), and floral symmetry are often suggested to be an adaptation by flowers to attract bumblebees. The models implemented here have been designed to produce predictions that can be tested using behavioural bumblebee studies.

The goal of this study is to a propose an information processing mechanism that can achieve unrewarded, unlearned symmetry preferences. We are extending our earlier work [18] by testing the hypothesis that inherent properties of visual signals are processed differently: some visual signals are computationally cheaper to process than others. We hypothesize that unrewarded preferences by bumblebees are related to the *computational cost* of processing floral signals. In other words, the compressibility of a signal is an indication of how easy it is to process information by a brain’s visual system. Our further assumption is that lower computational costs should translate into initial behavioural preferences. We are not suggesting that the brain must keep a faithful copy of the input and track its information processing cost. We are suggesting that bumblebees make a decision to land on a flower that carries lower information processing costs, and make a decision to not land on a flower that carries greater information processing costs.

The class of models that are ideal for evaluating this hypothesis are ones that are able to extract features from a high dimensional space and reduce it to a smaller feature space. Two unsupervised neural networks will be tested using visual stimuli that are identical to ones used in behavioural experiments investigating pattern shapes, pattern positions [17], symmetry, and spatial frequency [15]. By identical, we mean test stimuli were exactly the same: they were borrowed, with permission, from the authors that conducted the behavioural experiments.

## Models

The choice of models is greatly narrowed by the purpose of the study. Our goal is to simulate how cognitive features in an insect process visual signals to extract useful information. The model is not designed to be biologically plausible, but to capture the product of a particular cognitive function. More importantly, we are looking to quantify information processing in such a way that the results can be compared with empirical data. We are not looking to explain the way physical properties of electromagnetic waves are translated into electrochemical signals by the ommatidial array, nor are we looking to explain more advanced cognitive operations such as decision making, learning or memory related computations. There are numerous unsupervised non-linear neural networks that capture various aspects of low level visual processing with good results [19].

Our choice fell on point-models that attempt to capture key aspects of how visual information may be treated in an insect. We test two algorithms here, Independent Component Analysis (ICA), and Feature-Extracting Bidirectional Associative Memory (FEBAM) that detect local components in images of natural scenes with high performance. Both neural networks accomplish perceptual feature extraction in an unsupervised fashion to keep useful information and discard noise.

There are several differences between the models. ICA has visual signal processing roots, and is designed to work best with non-gaussian data. FEBAM and Bidirectional Associative Memory models have their roots in human memory research, and therefore, this breed of models is more general purpose. One particular difference in the mathematical basis between the two models lie in the form of signal they use: FEBAM deals with the second moment about the mean, covariance; ICA deals with the fourth moment about the mean, maximization of non-gaussianity.

### Input Signal

Input signal comprised of images of flower-like test patterns used in bumblebee behavioural experiments. Patterns evaluating hypotheses relating to radial, concentric and positioning of patterns used exact replicas of patterns used in [17]; symmetric and random patterns used exact replicas of patterns used in [20].

Bumblebees’ visual capabilities vary by the distance at which they view objects. Far objects (subtending at least 5° but not exceeding 15°) are only processed by green-channels (i.e., seen in grayscale), but nearby objects a processed by all three channels (i.e., seen in colour) [21, 22]. Based on hovering distance data [17], we are assuming that bumblebees make a decision to approach an object using grayscale information about the object. Extracting the green channel from each colour stimulus create grayscale patterns that vary on their level of lightness. For example, a blue stimulus will look darker than a yellow stimulus when converted to grayscale.

Pre-processing the image signal refers to a set of transformations that prepare the data for input into the networks. These steps include converting the original colour image into a grayscale image; cutting the image into small overlapping image patches; then converting the matrix representing an image patch into a vector, stringing all image patches into one long vector, and performing a Principal Component Analysis (PCA) on this long vector. We used PCA as a *whitening transformation* in which the variables become uncorrelated and have a variance of 1.0.

### The ICA Model

**Fig 1:**
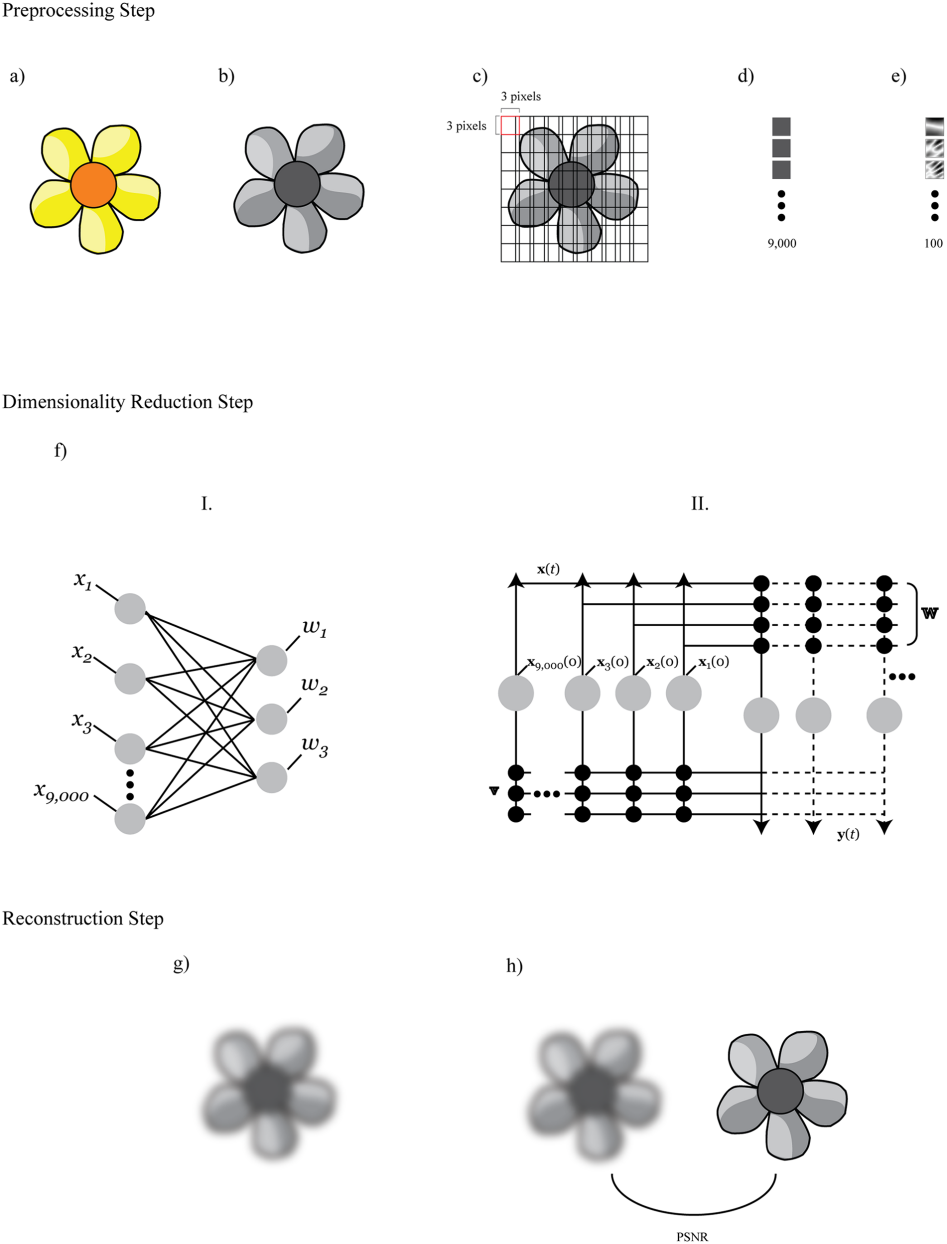
Summary of simulations and architecture of the models. First, original experimental images are loaded (a), then converted to grayscale (b), and sliced into highly overlapping image patches (c). The image patches are ordered into a long vector, which is then centered (d) and whitened (e) using Principal Component Analysis. The pre-processed signal is then input into one of the models (f): ICA (I.) or FEBAM (II.). At the end, the original image is reconstructed from the ICA or FEBAM features (g), and the quality of this reconstructed image is compared to the original using PSNR (h).

ICA can be performed on natural images by processing the observed signal in a statistical generative model, the components of which yield a representation of the original data [23]. The process is applied to centred [24] principal components of overlapping grayscale image patches, extracted from the chromatic source image. Fig 1 shows the architecture of the network.

ICA input mixture signal consisted of 3 × 3 overlapping grayscale image patches generated from a 100 × 100 pixel byte level image. The images were converted to grayscale using green-channel data to reflect how bumblebees might see a pattern with spatial resolution of 0.30–0.35 *cycles⋅degree* ^−1^ from a distance of 30 cm [21, 25–27]. There were a total of 9,000 overlapping image patches after this pre-processing step. After centering the image patch vectors, PCA was performed on each image to decorrelate the signal vectors. The principal components were fed into the deflation fixed-point ICA algorithm. This method uses *tanh* function to minimize mutual information, which tends to produce feature vectors in order of decreasing non-Gaussianity [28]. Independent components were generated using the fastICA algorithm, implemented in Mathematica [29]. The fastICA algorithm is described in more detail elsewhere [30].

Using the inverse of the estimation matrix ***W***, images used in behavioural studies were reconstructed. Here *x* refers to the test pattern or floral test images, and *y* is the matrix of statistically independent component vectors.

y=Wx (1)

Image ***x*′** was reconstructed using:

$$x^{\prime }=W^{Ty}$$ (2)

### The FEBAM Model

FEBAM is a modified version of Bidirectional Associative Memory (BAM) [31, 32] where one set of connections was removed. This modification makes the network act as an unsupervised associative memory. Fig 1 shows the architecture of this model:

The **W** weights send information to the output layer, and the **V** weights send information back to the x-layer in a top-down bottom-up manner. A detailed description of this model is found elsewhere [31]. Activation is expressed by the following relations:

$$y\;\left(t+1\right)=g\;(\left(\delta +1\right)Wx\;\left(t\right)-\delta \;{\left(Wx\;\left(t\right)\right)}^{3})$$ (3)

$$x\;\left(t+1\right)=g\;(\left(\delta +1\right)Vy\;\left(t\right)-\delta \;{\left(Vy\left(t\right)\right)}^{3})$$ (4)

where *W* and *V* are weight matrices, *y* refers to the distributed filters (i.e., feature vectors) across the units, *x*(0) is the original image input, and the reconstructed image is *x*(t+1). *δ* is a general output parameter that determines the type of attractor the network will exhibit (fixed-point, cyclic, chaotic). This value was held constant in this implementation at 0.1 to produce fixed-point attractors. The output of the piecewise function *g* behaves like a sigmoid-type function, but without the asymptotic property:

$$g\;\left(z\right)=\{\begin{array}{ccc} +1, & \text{If} & z>1\\ -1, & \text{If} & z<-1\\ & \text{Else} & z \end{array}$$ (5)

The images were also pre-processed in the same manner as in ICA: images were converted to grayscale, centred, and principal components extracted. Images were reconstructed using the ***W*** and ***V*** matrices.

### Outcome Measures

The quality of the reconstructed image was compared to the original image using the Peak-Signal-to-Noise-Ratio (PSNR) measure [33]. The rationale for choosing this measure follows our assumption that reconstruction quality of images may indicate the cognitive cost to process the test patterns. We kept all parameters constant across simulations, so better quality of reconstruction means that a fixed number of feature vectors (i.e., 3 vectors in ICA and in FEBAM) captured more relevant information to reconstruct the image. Therefore, we suggest that the inherent characteristics of test patterns are computationally more affordable to process and thereby should be more preferred by pollinators, provided prior reward has not been associated with any other visual property. In concrete terms, lower PSNR values map onto poorer recognition whereas higher PSNR values correspond with better recognition of stimuli. Precise differences in PSNR values are known to correspond with perceptual differences in humans (e.g., as little as 0.5 dB can be detected), but the relationship between precise PSNR values and bumblebee choice behaviour have not been studied.

## Results

Three categories of images were used. First, test patterns manipulating pattern positioning and pattern type, which mimic ones used in our behavioural experiment [17]. Second, images of background and artificial floral stimuli, which mimic ones used by Forrest et al [16]. Third, test patterns manipulating symmetry and spatial frequency were generated using the same algorithm that generated patterns for a behavioural experiment [15]. All simulations used Monte Carlo sampling method and performed 300 times.

### The *Nectar Guide* hypothesis: Radial vs concentric patterns

**Fig 2:**
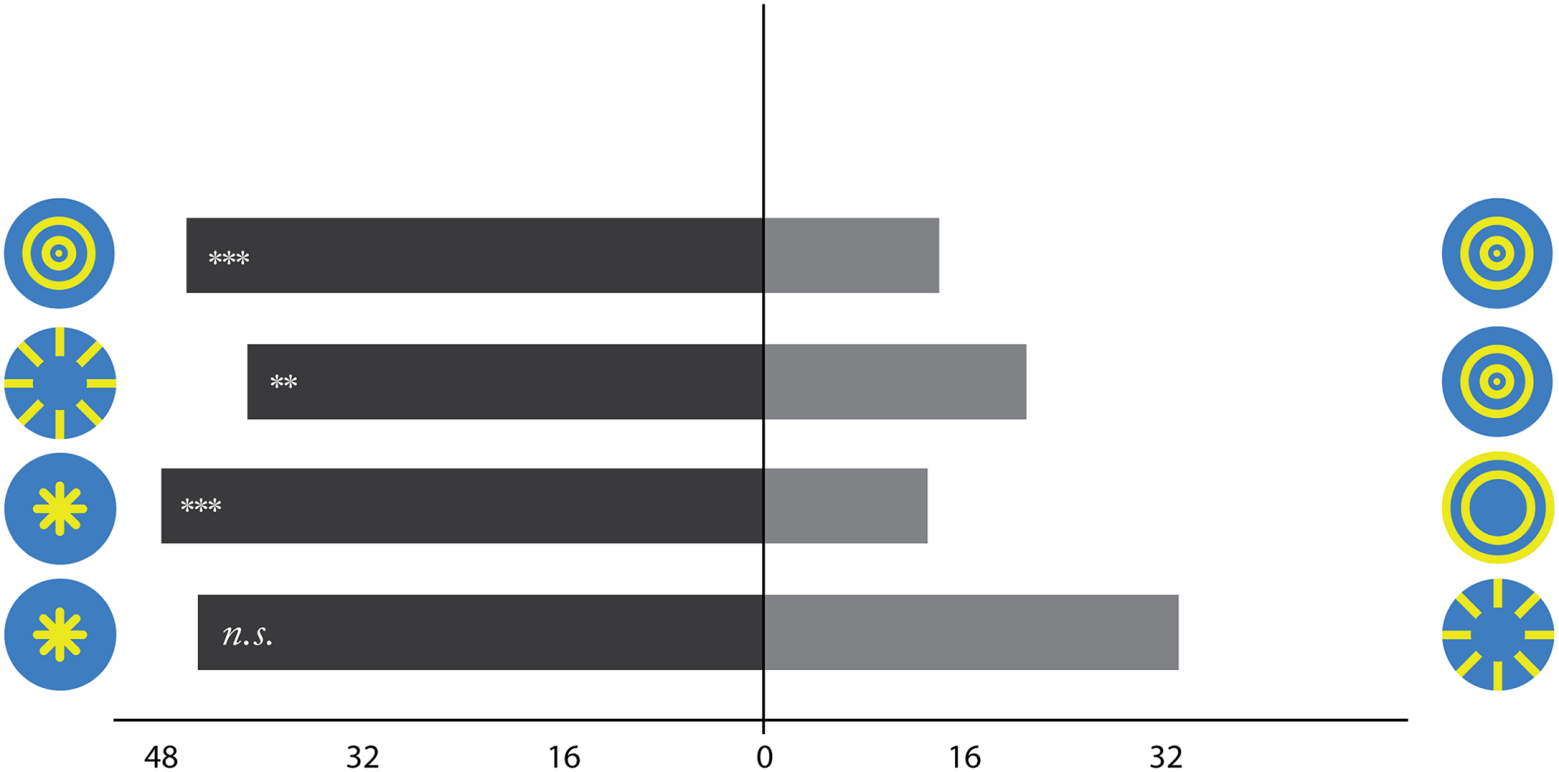
Behavioural pattern preferences: Pattern positioning *versus* pattern type. This bar graph shows the total number of choices made by bumblebee workers at each pattern combination. Whenever a radial pattern was pitted against a concentric one, bumblebee workers chose the radial pattern. In the last case where two radial patterns of different positioning were displayed, bumblebees showed no preference for either pattern. Adopted from Orbán & Plowright [37] *Note*: ** <.01, *** *p* <.001, *n*.*s*.: not significant. The figure shows test patterns in their original form, as used in the behavioural experiment.

Many behavioural studies document an unlearned preference for radial patterns (ie, sunburst pattern) over concentric patterns (ie, bull’s eye pattern) and over other shapes not typically found in nature [13, 14, 17, 34, 35]. For example, Orban et al [17], displayed radial and concentric patterns to free-flying flower-naïve *B. impatiens* workers where the appearance of the pattern was manipulated to be visible only at the centre or only at the periphery of artificial flowers. Flower naïve means that the bumblebees left the nest for the first time and have never received any reward in the testing environment. The results showed that regardless of positioning, workers preferred to land on patterns with radial shapes (See Fig 2). Other studies have also shown similar effects for radial patterns [13, 14, 34, 35]. The rationale originates in the 19th century when Sprengel suggested that “honey guides” help honeybees discover the source of rewards [36].

**Fig 3:**
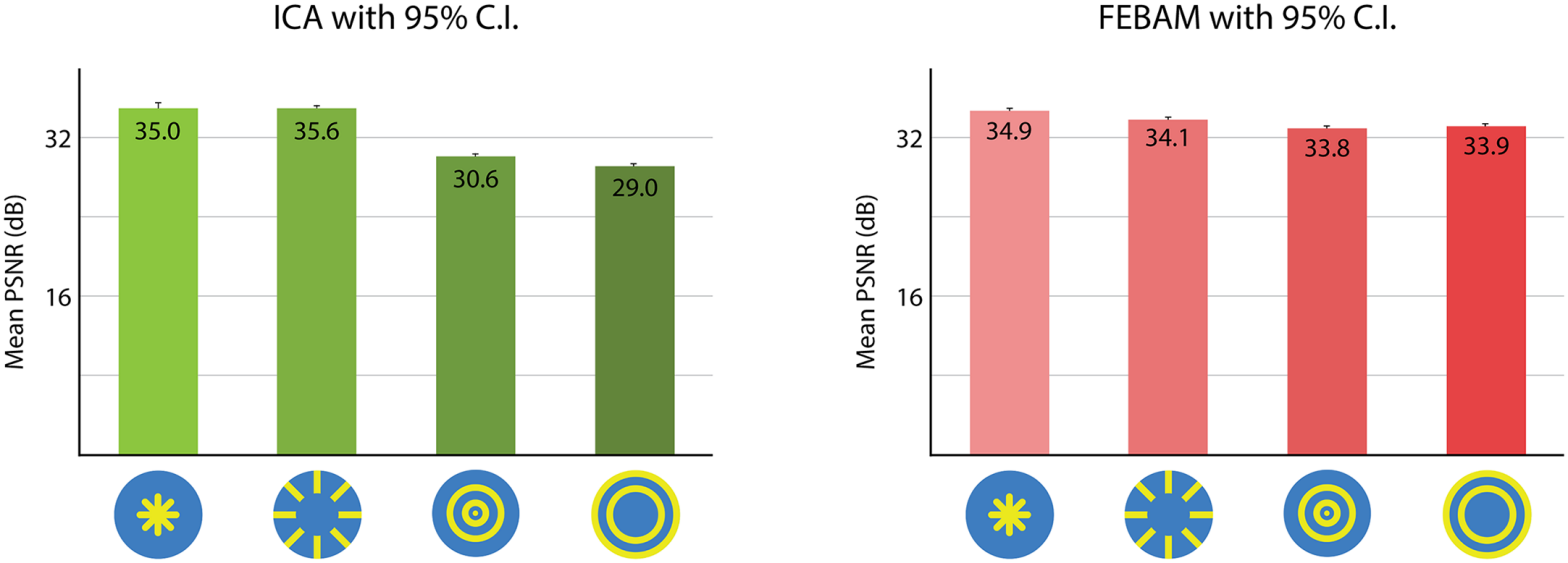
ICA & FEBAM model results for pattern positioning and pattern type. PSNR values for pattern type and positioning. Each pattern in the figure was reconstructed using its own filters generated by either the ICA or FEBAM models, and the quality of this new image was compared to the original image. Results are based on 300 simulations. Error bars show 95% confidence interval. The test patterns in the figure were used in a behavioural experiment elsewhere.

ICA and FEBAM networks show similarities and differences: overall, radial patterns are better reconstructed than concentric patterns (see Fig 3). Best quality reconstruction is achieved by the peripheral radial pattern using ICA, and the central radial pattern using FEBAM. In between are the peripheral radial pattern and the central concentric pattern. The quality differences in FEBAM are very small, but ICA shows substantial quality differences that correspond with behavioural findings. More specifically, the central and peripheral radial patterns show a statistically non-significant difference, which matches behavioural findings. Central and peripheral concentric patterns are each reconstructed in significantly lower quality, consistent with behavioural findings.

### Symmetry and Spatial Frequency

Six kinds of simulations were performed: asymmetric (ie, random), 1-axis (ie, bilateral) and 4-axis symmetric patterns in low and high spatial frequency variations were used. Low spatial frequency was generated to have 4,000 pixels, and high spatial frequency to have 9,000 pixels of black and white perimeter. Perimeter lengths were verified using an edge detection and pixel counting algorithm and allowed to vary by up to 5%. Further, area of black and white was set to 50% and allowed to vary by up to 5%.

#### 1-axis symmetry

**Fig 4:**
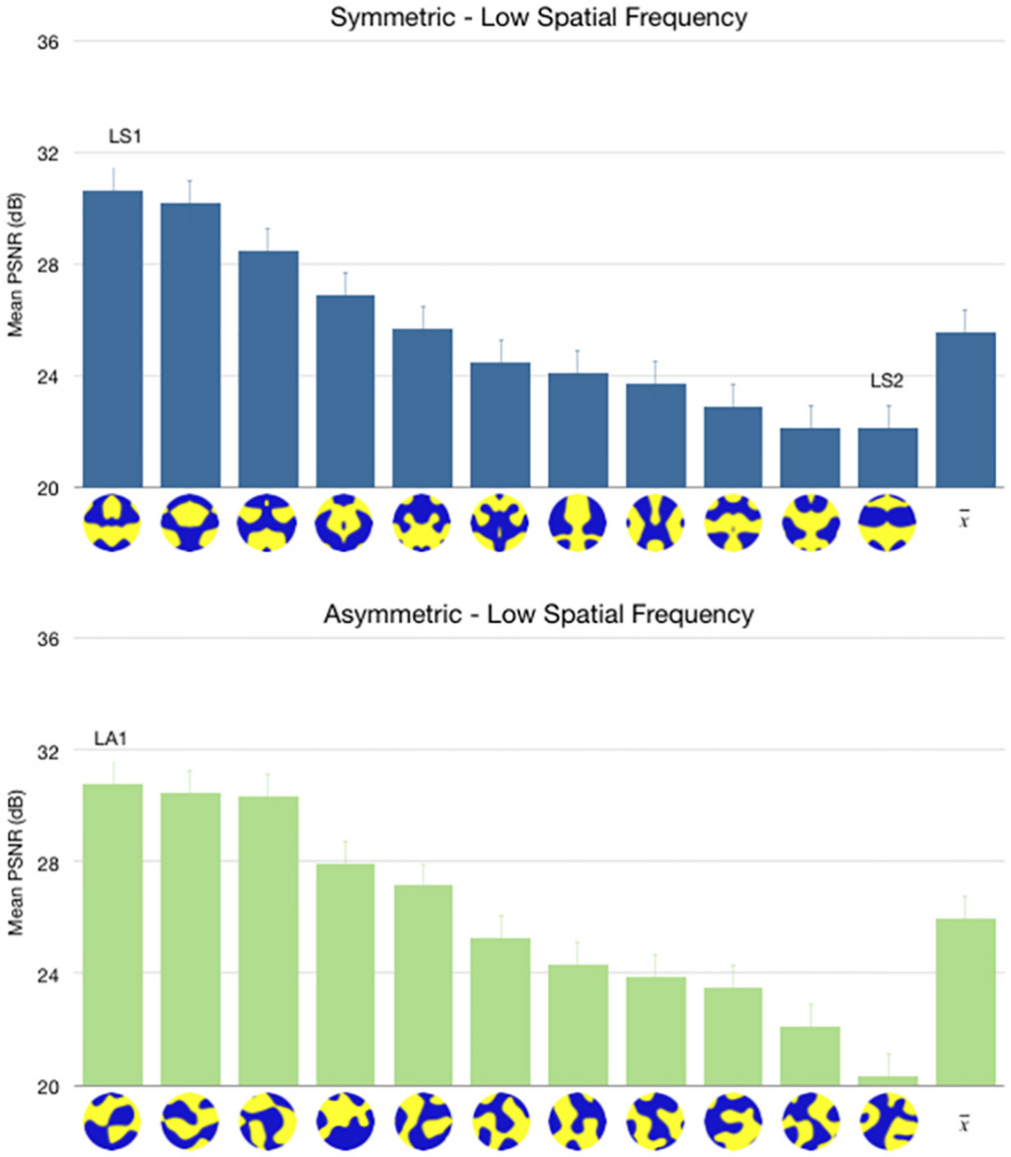
PSNR reconstruction values for low spatial frequency symmetric and asymmetric patterns. Test patterns are ordered from best PSNR values to worst PSNR values, and the last bar shows the mean PSNR value for all test patterns of the same category. Results are based on 300 simulations. Error bars show 95% confidence interval. The patterns labelled “LS1” and “LA1” were used in a behavioural experiment testing the predictions of this model, reported in [38].

**Fig 5:**
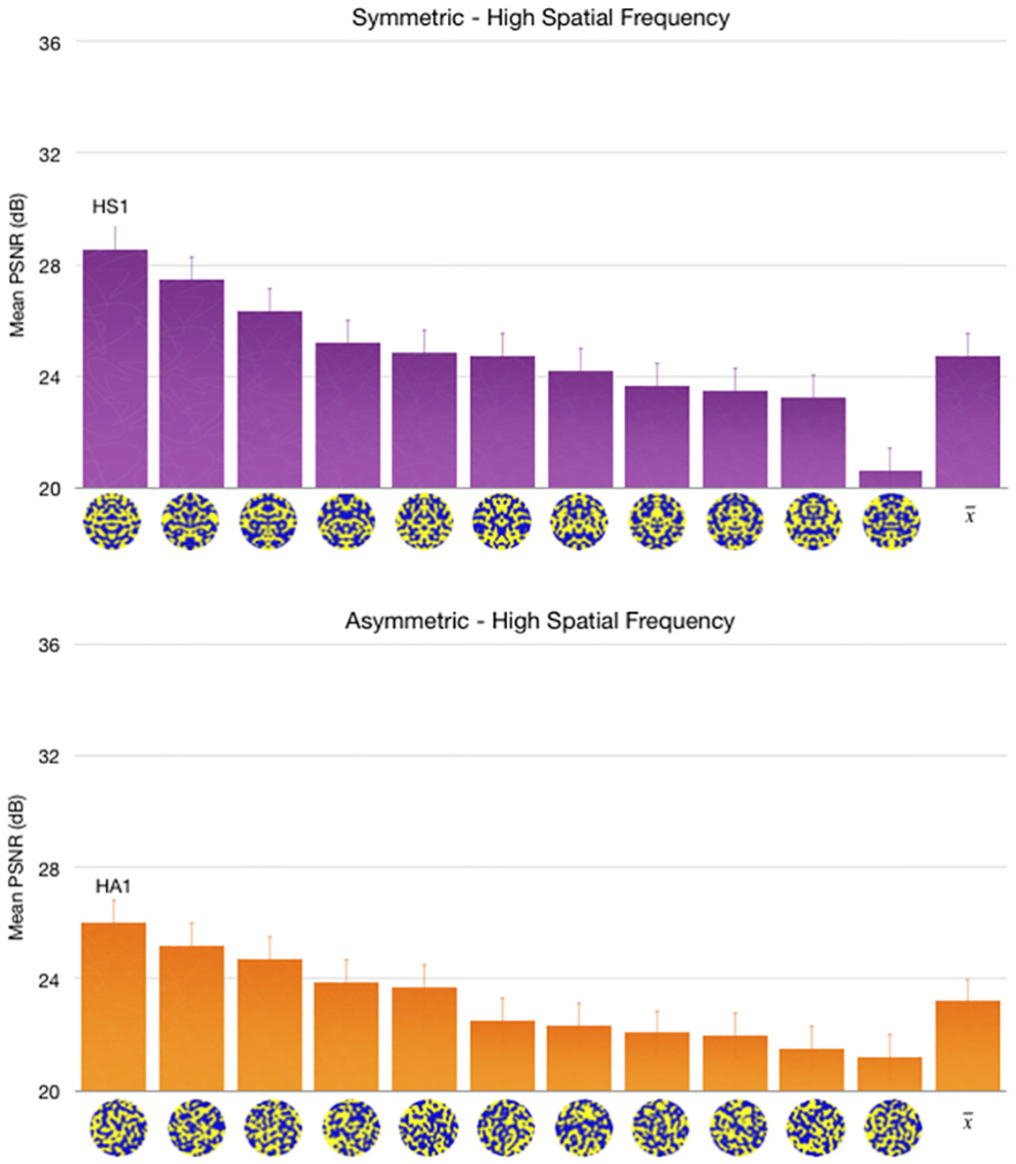
PSNR reconstruction values for high-spatial frequency symmetric and asymmetric patterns. Error bars show 95% confidence interval. The patterns labelled “HS1” and “HA1” were selected for a behavioural experiment testing the predictions of this model. The figure shows test patterns in their original form, as used in the behavioural experiment.

ICA and FEBAM models produce corresponding but unexpected results when we manipulated symmetry and spatial frequency (See Figs 4 and 5). The test pattern of high spatial frequency show better reconstruction for the bilaterally symmetric pattern when compared with high spatial frequency asymmetric pattern. However, the low spatial frequency patterns do not show this trend: the asymmetric and symmetric patterns had comparable reconstruction qualities.

#### Four-axis symmetry

**Fig 6:**
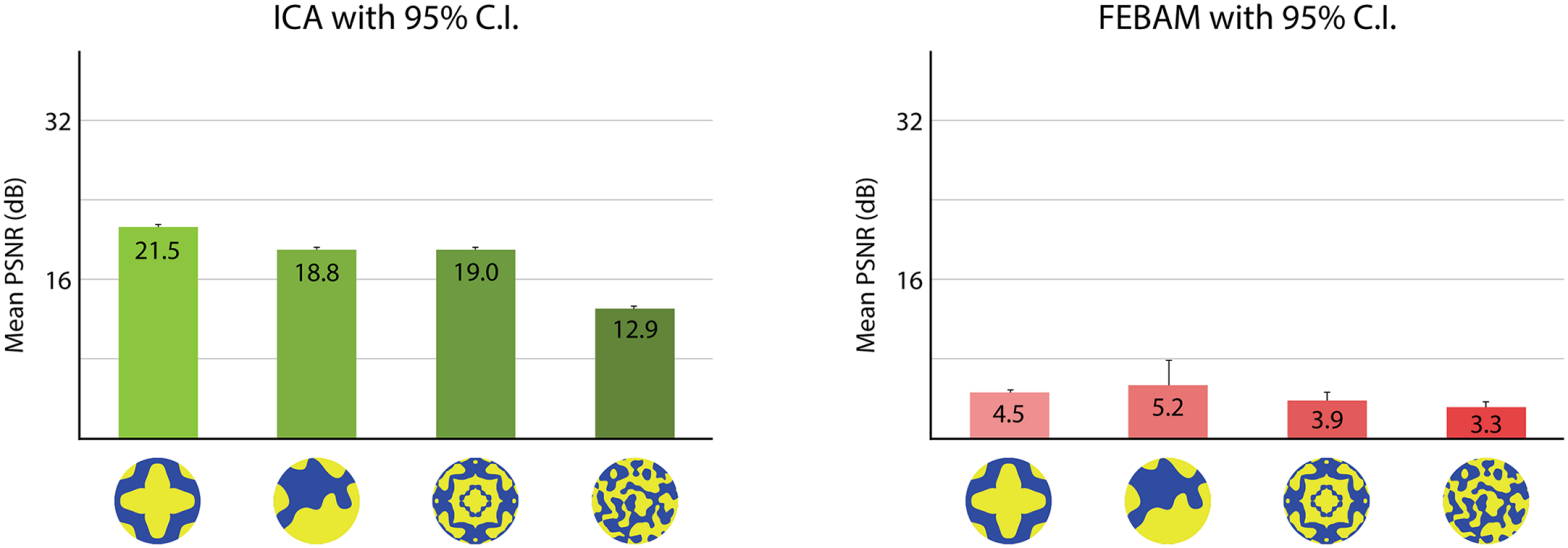
PSNR reconstruction values for 4-axis symmetric and asymmetric patterns. Error bars show 95% confidence intervals. The figure shows test patterns in their original form, as used in the behavioural experiment.

The increased redundancy in 4-axis symmetric patterns compared to bilaterally symmetric patterns resulted in a substantial change in quality of image reconstruction. The effect of symmetry is now observed even in the low spatial frequency domain. Four-axis symmetric patterns were significantly better reconstructed regardless of spatial frequency (See Fig 6).

#### Spatial Frequency

**Fig 7:**
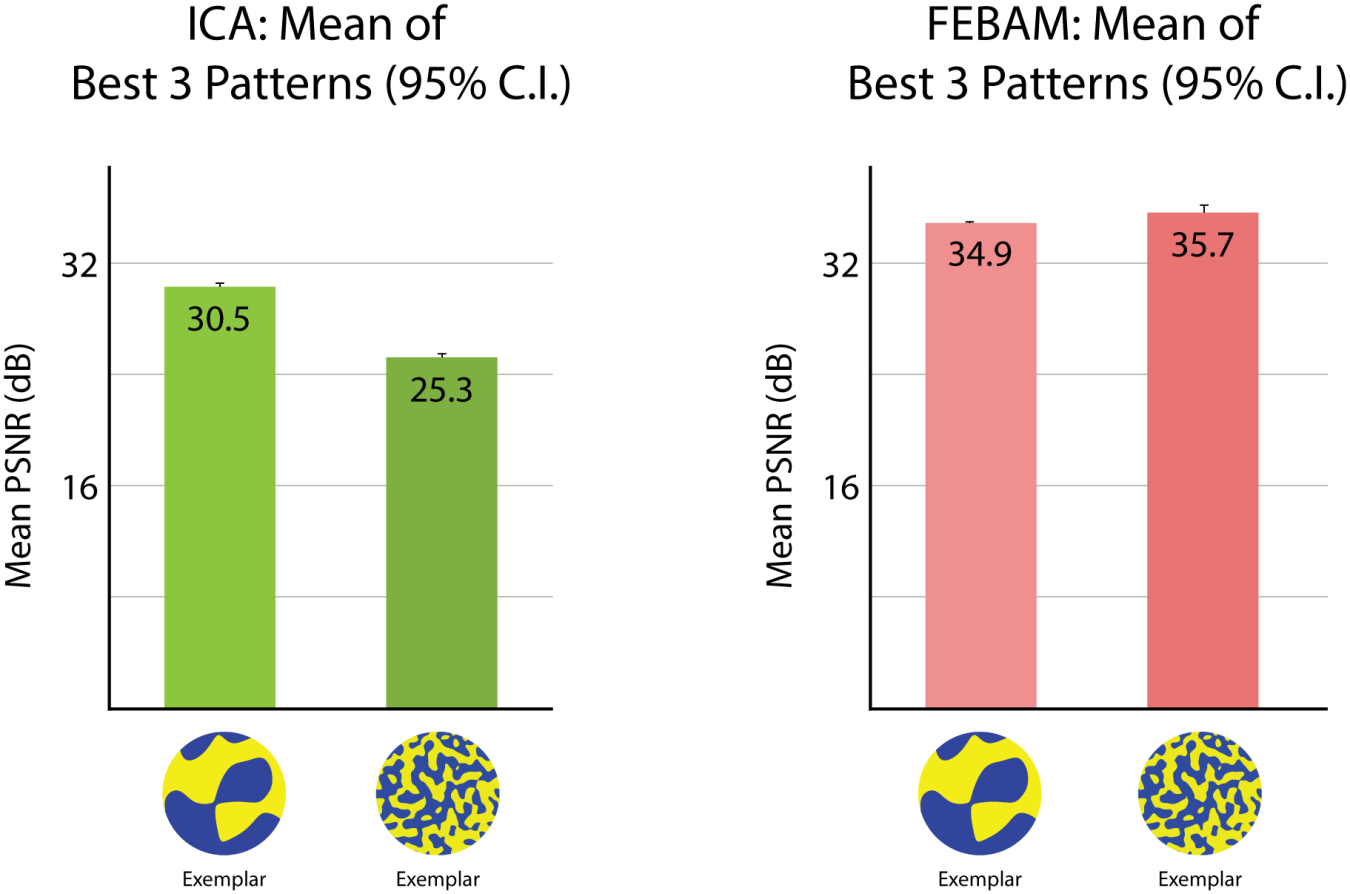
PSNR reconstruction values for high and low spatial frequency asymmetric patterns. Results are based on the three patterns reconstructed in best quality (i.e., left-most patterns from Figs 4 and 5), each pattern simulation performed 50 times. Error bars show 95% confidence interval. The figure shows test patterns in their original form, as used in the behavioural experiment.

FEBAM and ICA diverge in results relating to spatial frequency. ICA captures behavioural findings that indicate a preference for low spatial-frequencies, though spatial-frequency was never specified beyond “low” or “high” (see Fig 7). FEBAM shows the opposite result where high spatial frequency patterns were reconstructed in better quality.

## Discussion

Both unsupervised non-linear neural networks show a consistency with behavioural results of unlearned floral choice by bumblebees and honeybees. The results of ICA are more robust because this model captured more subtle findings related to floral positioning, and more consistency for findings related to spatial-frequency. This may be an indication that the ICA model captures a key information processing element in the way these pollinators process and respond to visual information. The results lend support to the idea that bumblebees and honeybees show a behavioural preference toward the tested patterns as a result of a by-product in their visual information processing systems.

### Consistency with behavioural studies

Our findings with respect to symmetry and spatial frequency produced two testable hypotheses. First, low frequency symmetric patterns should be preferred over high-frequency symmetric patterns, and even more interestingly, an interaction between symmetry and spatial frequency should be observed. The interaction effect may indicate a ceiling in the amount of information that bumblebees’ and honeybees’ visual system can code.

This is a novel prediction ready for empirical observation: behavioural studies have been mixed in terms of symmetry preferences. For example, Rodríguez et al, [39] found innate bilateral symmetry preference by *B. terrestris* workers, but others have not found such a preference [15]. Likewise, the computational models of Enquist et al, [40] and Johnstone et al, [9] imply that bilateral symmetry preference may be a learning by-product of the visual system or a born-with capacity.

In a separate behavioural experiment, we tested and confirmed the predictions of the ICA model with *B. impatiens* workers [38]. We pitted against the two visual properties of symmetry and spatial frequency using a free flying choice paradigm. Symmetry was presented at three levels, at 0-axis (random), 1-axis and 4-axes of symmetry, and spatial frequency was presented at two levels (low and high). The choice of bumblebee workers was significantly influenced by spatial frequency in the 1-axis symmetry condition, and consistently symmetric patterns were chosen in the 4-axes symmetric condition.

### Differences between ICA vs FEBAM

Differences in the performance of ICA and FEBAM may lie in how features are extracted: FEBAM is based on the second moment about the mean, covariance, while ICA maximizes of non-guassianity, which is the fourth moment about the mean. Neuroscientific data indicate that covariance information may be useful when receiving redundant information from multiple organs of the same modality. For example, the Jeffress model suggests that the auditory system uses covariance structure to extract Interaural Time Difference in order to localize the source of stimuli in space [41, 42]. On the other hand, one of the features of many natural signals, including visual signals, is that they are non-gaussian. Perhaps using non-gaussianity to extract features corresponds with the visual signal better than covariance. ICA also shows correspondence with the way visual receptive fields self-organize with principles of decorrelation and sparseness. Visualization of ICA filters have been likened to edge detectors in V1 of the primary visual cortex [43]. This may account for why ICA consistently outperformed FEBAM in terms of capturing behavioural results.

### Model comparison

Enquist et al, [40] and Johnston et al, [9] also proposed computational models that suggested unlearned preference to be a by-product of the visual system, but with two key differences: (1) one model focusses on the how unlearned preference for symmetric patterns could emerge over several generations, and (2) the other model focusses on how functional experience with flowers can generate preferences for symmetry.

Here, we propose a mechanism by which unlearned symmetry preferences could emerge due to particular information processing characteristics. The results here support the feedforward genetic algorithm by proposing an information processing mechanism by which unrewarded symmetry preferences could emerge, and extend this work to other kinds of visual properties, including pattern, colour and positioning. What makes our model novel is that it captures behavioural results across several visual properties.

The aim of this study was to test the idea that the computational cost of processing stimuli could be measured in such a way that it excludes the metabolic cost of information processing. The rate of information processing under different levels of light intensity has been measured, but it is not clear how much of this cost is due to the maintenance of the “processor” or the brain, and how much is due to the cost of processing the information. Here we suggest that the use of unsupervised neural networks may be used to estimate the cost of information processing minus the cost of metabolic processes. The accuracy of this model for estimating computational cost depends on the extent to which it captures behavioural data.

While we used image reconstruction as a proxy for computational cost, this does not necessarily mean that bumblebees reconstruct the image somewhere in their brains. Reconstruction is just a mean for the experimenter to see how well the algorithm is performing. In the biological system, the compressed information extracted from the components would bias choice, among several other potential outputs.

### Future work

Several aspects of the models could be adjusted to capture different visual properties or increase the precision of the current results. Spatially-sensitive unsupervised neural networks such as Self-Organizing Maps [44] and Topological Bidirectional Heteroassociative memory models could be a potential candidate for future simulations [45]. ICA and FEBAM are point-models where features are not easily ranked or prioritized in terms of importance. The spatially-sensitive models may uncover a subset of features that are especially important, and may also reveal relationships between features that are not visible from the current models.

In this model we reconstructed the images and compared them to the original version using PSNR values. Future models may include Bayesian detection and classification instead. The neural networks could also be further tuned to simulate the physiological properties of different pollinators’ visual systems. For example, instead of using byte level information from the image, summarizing independent channels using histogram information would be a statistically more meaningful representation [46].
